# Subjective wellbeing and psychological symptoms of university students during the COVID-19 pandemic: Results of a structured telephone interview in a large sample of university students

**DOI:** 10.3389/fpsyg.2022.889503

**Published:** 2022-09-15

**Authors:** Imke Baetens, Johan Vanderfaeillie, Veerle Soyez, Tim Vantilborgh, Joyce Van Den Meersschaut, Chris Schotte, Peter Theuns

**Affiliations:** BRUCC Research Group, Faculty of Psychology and Educational Sciences, Vrije Universiteit Brussel, Brussels, Belgium

**Keywords:** wellbeing, university students, psychological distress, COVID-19 pandemic, mental ill-being

## Abstract

University students are at elevated risk for psychological distress, especially during the COVID-19 pandemic. The aim of this study was to warmly contact our students and investigate the psychological impact of the COVID-19 pandemic on the subjective wellbeing (SWB) and levels of psychological symptoms (such as depressive and anxious feelings) of university students in Belgium. All bachelor and master students of the Vrije Universiteit Brussels (*N* = 15,475) were invited for a brief structured telephone interview in March, 2021. In total, 7,154 students were assessed by a structured interview, based on the Kessler Psychological Distress Scale (K6) and the Anamnestic Comparative Self-Assessment (ACSA). Compared to a representative sample, students considered their life during the pandemic as less satisfying compared to their life before the pandemic. Overall, all students have suffered from COVID-19 and the measures taken to contain the pandemic. Twenty percent of our sample of 7,154 VUB students scored above the K6 cutoff, indicating a heightened risk for having a diagnosable mental illness severe enough to cause functional limitations and to require treatment. This study highlights the need for psychological support for all students, during the COVID-19 pandemic.

## Introduction

The COVID-19 pandemic outbreak has caused global and unprecedented challenges for mental health (Iob et al., 2020; Varga et al., 2021). The pandemic and its control measures, such as travel restrictions and social and physical distancing measures have substantially affected the international higher education sector. All over the world, university campuses were closed, and activities were postponed, while courses moved to online platforms. A growing body of international research shows a global decrease in mental wellbeing and an increase in psychopathology at the population level and an increased risk for especially adolescents and college students (e.g., Naser et al., 2020; Varga et al., 2021). For example, in Belgium, rates for severe psychopathology increased from 1.5% at the beginning of the first lockdown in March 2020 to 6% 3 months later (Neyens et al., 2020).

In this study, we examine the psychological impact of the COVID-19 pandemic for students enrolled at the Vrije Universiteit Brussel. We start with a literature review regarding the impact of the COVID-19 pandemic and the restrictions to contain the pandemic on the functioning of university students. Next, we focus on the methods used and results found. Data on perceived subjective mental wellbeing of university students and the prevalence and severity of psychological symptoms will be presented. Finally, a discussion and conclusion are formulated.

According to the definition of the World Health Organization, good mental health is “*a state of complete wellbeing and not merely the absence of disease or infirmity*” (WHO, 1946 in World Health Organization, 2006). Moreover, the World Health Organization (2001) states that “*without mental health and a sense of wellbeing, there is no real health.*” Consequently, examining the impact on mental health of both the COVID-19 pandemic and the measures taken to contain this pandemic, should not be limited to the increase of psychological symptoms as key mental health indicator. Rather, a combination of indicators situated on both dimensions of a two-factor model of mental health need to be considered (Keyes, 2005). This model distinguishes positive subjective wellbeing and psychopathology, as separate but related constructs (Keyes, 2005; Suldo and Shaffer, 2008; Westerhof and Keyes, 2010). The extent to which someone experiences psychological distress, or has a disorder, does not necessarily provide more insight into their subjective wellbeing. Several studies show that subjective wellbeing can vary greatly between people with a same psychological disorder or symptoms (e.g., Teismann et al., 2017). Psychological distress is used as a widespread indicator of mental health and mental illness, both in research and clinical settings (Drapeau et al., 2010). It combines mostly symptoms of depression and anxiety, which are considered indicators of an intense feeling of emotional ill-being.

COVID-19 and the measures taken to contain the pandemic had a detrimental impact on subjective wellbeing (Nelson et al., 2020). A prospective cohort study among United Kingdom university students showed a significant drop in subjective wellbeing over four time points (the first two before and the last two during a lockdown), with a medium effect size (Savage et al., 2020). Also, the fifth national Belgian health survey (Vijfde COVID-19-gezondheidsenquête, 2020) reported a significant drop in subjective life satisfaction during the COVID-19 period. Although this decrease is observed in all age groups, nearly 50% of young adults report dissatisfaction with their current life. The aforementioned studies all suggest that a lack of direct social interactions with peers leads to increased loneliness, low perceived social support and social isolation, thus decreasing subjective wellbeing (Hamza et al., 2021; Pieh et al., 2021a). Dodd et al. (2021) reviewed conceptualizations and measurements of well-being in UK university students and highlighted multiple inconsistencies in defining and measuring university student’s well-being. Therefore, the present study uses the ACSA (Bernheim et al., 2014) and two conventional single item rating scales to measure global subjective well-being. Keyes (2006) describes subjective wellbeing as the perception and evaluation of one own life.

Several national and international studies have also highlighted an increase in psychological distress since the COVID-19 outbreak, especially in student populations (e.g., Naser et al., 2020; Neyens et al., 2020; Mack et al., 2021). Symptoms of anxiety and depression are most visible in 18–25-year-old (Vijfde COVID-19-gezondheidsenquête, 2020; Mack et al., 2021). For example, it was found that about two-thirds of young adults experienced mild psychological symptoms (e.g., anxious feelings) during the first lockdown (Rens et al., 2021). A longitudinal study in college students (18–24 years) in April, June, and September 2020 found a consistently high prevalence (about 30%) of depressive symptoms (Pieh et al., 2021a,b). During the second lockdown in December 2020 (in Austria), the prevalence of depressive symptoms even increased in this college population to up to 50% (Dale et al., 2021). In a large-scale meta-analysis of 11 longitudinal studies, it was found that psychological distress increased from pre-pandemic to peri-pandemic (Patel et al., 2022). High levels of psychological distress (i.e., indicators for diagnosable psychopathology) increased over the three timepoints from March 2020 to October 2020 to March 2021. Overall, rates of psychological symptoms increased over these three COVID timepoints. Essau and de la Torre-Luque (2021) examined levels of severe psychological distress (using the K6 questionnaire; Kessler et al., 2002) in participants from the Millennium Cohort Study (MCS; Connelly and Platt, 2014), aged 19 in the first lockdown and reported that 23% of the 900 participating young adults reported severe psychological distress. Previous studies have consistently shown that about 50% of mental disorders emerge in late-adolescence and young adulthood (Jones, 2013).

Pre-COVID studies found that female students and freshmen are at heightened risk for severe psychological distress. Also, during the COVID pandemic, several studies show that females are at higher risk to suffer from the COVID-19 pandemic and the measures to contain it (Kecojevic et al., 2020; Rens et al., 2021; Visser and Law-van Wyk, 2021). Especially college students in their early years of study seem most at risk of experiencing emotional difficulties, although some scholars report opposing findings. In the sample of Kecojevic et al., 2020, for example, non-freshmen (sophomores, juniors, and seniors) were found to be at higher risk for psychological symptoms compared to freshmen. Super and Van Disseldorp (2020) found that master students reported more anxiety symptoms than bachelor students, whereas bachelor students were more likely to report depressive symptoms and feelings of loneliness. Moreover, as was pointed out by Chen et al. (2020) international students have faced more impediments with regards to mental health and wellbeing during the COVID-19 pandemic opposed to local students.

Although a consistent body of literature over the past decade (e.g., Schwenk et al., 2010) indicates that medical students are at high risk for psychological symptoms, little is known about differences in risk for psychological symptoms during the COVID-19 pandemic across faculties and programs. One study (Kohls et al., 2021) has reported that during the COVID-19 pandemic especially students in the Humanities scored the highest on psychological symptoms (i.e., depressive symptoms and suicidal ideation), whereas students from medical faculties scored the lowest. Further, there is little consensus in the literature on the impact of the COVID-19 pandemic on social wellbeing (SWB) and psychological distress across different faculties and programs, which can be identified as an important gap in the research.

The current study presents findings on the prevalence and severity of psychological symptoms and subjective wellbeing in university students in Belgium during the COVID-19 pandemic (March 2021). In line with Savage et al. (2020); we expect a significant drop in subjective wellbeing. Consistent with national and international studies (e.g., Dale et al., 2021; Rens et al., 2021) we expect more mild psychological symptoms, and an increase of severe psychological distress. Based on previous studies, we will examine associations with relevant socio-demographics (gender, academic standing, and age). In line with Rens et al. (2021), we expect female university students to be at the greatest risk for decreased subjective wellbeing and increased psychological distress. Basing on Kecojevic et al., 2020, we expect that younger age is a risk factor for increased impact of COVID-19 on subjective wellbeing and levels of psychological distress. Also, we examined if international students are at greater risk for decreased subjective wellbeing and increased levels of psychological distress in line with Chen et al. (2020). In accordance with recent studies (Kohls et al., 2021), we expect students in the Humanities to report more psychological distress than students from other faculties.

## Materials and methods

### Participants

The aim of this project was 2-folded: First and foremost, during the COVID-19 pandemic, our university wanted to organize a warm phone contact with all VUB students individually, to let them know how VUB cares for them. Second, given this occasion, we wanted to objectively report on how much students were struggling and how many of them were at heightened risk for psychological distress. Therefore, we used validated measures suitable for a brief structured telephone screening. As a consequence of these aims, this study concerns a naturalistic uncontrolled sample.

**Figure 1:**
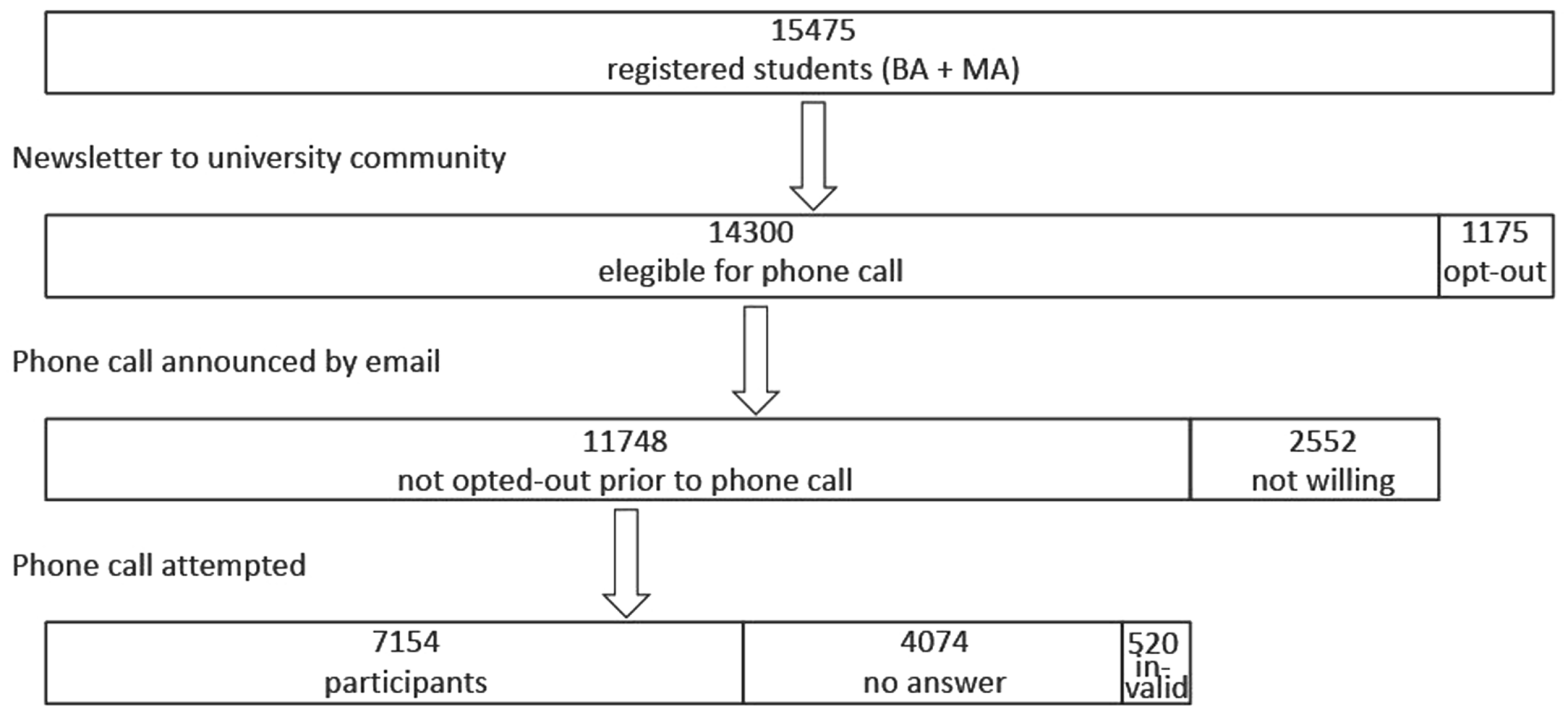
Flowchart of the sample. The monthly VUB newsletter which is also sent to all bachelor and master students registered at the Vrije Universiteit Brussel announced the phone call initiative. Students could opt out *via* an external link or by sending an email. Those who did not opt out were eligible for a phone call. These students were contacted by email, saying they would be called for a telephone interview. Some students replied to this mail, saying they were not willing to participate. Next, a phone call was attempted to the remaining 11,748 students. In the end there were 7,154 participants, 4,074 did not answer (three attempts), and 520 phone numbers were invalid.

PhD students, students in a post-graduate training and exchange students were excluded from this study. In February 2021, all bachelor and master students enrolled at the Vrije Universiteit Brussel (*N =* 15,475 registered students), were informed about the phone call initiative in a university newsletter and an email. Both announcements indicated how students could opt out of being called (1,175 opted out following the newsletter, 2,552 indicated not willing to participate in reply to the email). The remaining 11,748 students were then called by 134 trained and supervised master students in psychology. There were 520 invalid phone numbers, 4,074 students could not be reached in three attempts, so that 7,154 students actually participated. Figure 1 provides information on the recruitment procedure, response rates, numbers of opt outs, and non-response. All participating students were contacted for an interview by phone between March 1, 2021 and March 31, 2021.

Sample characteristics of the 7,154 participating university students (i.e., a 46% overall response rate; 59.4% females) are presented in Table 1. The comparison of the participants to the total student population (of bachelor and master students) indicates that the sample corresponds to the total student population.

**Table 1:** Sample characteristics (*N* = 7,154, for 35 students no socio-demographic information is available) compared to the total student population (*N* = 15,475, enrolled in Bachelor or Master programs, excluding PhD students).

	Count	Percent in sample	Percent in university
*Gender*			
Female	4,247	59.4	58.5
Male	2,872	40.1	41.5
*Age*			
< 18 years	9	0.1	0.2
18–20	2,459	34.4	32.5
21–25	3,444	48.1	47.5
>25 years	1,242	17.4	19.8
*Nationality*			
Belgian	6,083	85.0	82.5
Other EU country	424	5.9	7.2
Non-EU country (including United Kingdom)	612	8.6	10.4
*Work status*			
No job	5,458	76.3	74.5
Employed or self-employed	1,522	21.3	23.4
Alimony	139	1.9	2.1
*Academic standing*			
Freshmen (first year BA student)	1,178	16.5	15.3
Sophomore/Junior (other BA student)	3,219	45.0	45.2
Master	2,722	38.0	39.5
*Faculty*			
Medicine and Pharmacy	638	8.9	9.1
Engineering Sciences	481	6.7	6.8
Literature and Philosophy	524	7.3	7.7
Physical Education & Physiotherapy	429	6.0	5.8
Psychology and Educational Sciences	979	13.7	12.9
Law and Criminology	1,119	15.6	15.1
Social Sciences and Solvay Business School	2,136	29.9	31.0
Faculty of Sciences and Bioengineering	701	9.8	9.7
Teacher Education	112	1.6	1.9

### Measures

Next to some open-ended questions (such as “How are you doing?”) and some socio-demographic characteristics, the following questions and questionnaires were administered: three items inquiring subjective wellbeing [two conventional rating scale questions on satisfaction with life: one referring to the COVID-19 period and the other referring to the past 30 days, and the Anamnestic Comparative Self-Assessment (ACSA; Bernheim et al., 2014)]. Psychological distress was assessed with the K6 Psychological Distress Scale (Kessler et al., 2003).

#### Socio-demographics and academic status

Collected data comprised: gender, age, nationality (Belgian, other EU countries, and non-EU countries), and academic standing coded into three categories: Freshmen (students who enrolled for the time ever in a first year bachelor program), Sophomore/Junior (non-Freshmen taking only courses at bachelor level), and Master. The Vrije Universiteit Brussel has eight faculties and a separate program for teacher education that is here considered as a ninth faculty.

#### Subjective wellbeing

Anamnestic Comparative Self-Assessment (ACSA) is a self-anchoring rating scale for subjective wellbeing that was originally developed for use with cancer patients to address the problem that common measures of SWB were found insensitive to objective changes in the patients’ condition over time (Bernheim, 1983), likely caused by the changing of the frame of reference that patients use when assessing their subjective wellbeing, that is, relative to their current frame of reference (where healthy people would compare themselves to other healthy people and sick people would compare their own condition to that of other sick people, so causing a “response shift”; Schwartz et al., 2007). To overcome such response shift, Bernheim instructed patients to use a same idiographic frame of reference in successive assessments. Therefore, patients were invited to identify the best and worst periods in their lives and to assign, respectively, anchor ratings +5 and −5 to these periods. Further, assessments of SWB (typically concerning the last 2 weeks) would then be made relative to this personal −5 to +5 scale (Bernheim, 1983, 1999; Bernheim et al., 2006, 2014). The use of the ACSA was proven to be useful also outside clinical settings where it originated, such as education (Verhofstadt et al., 2019), and community studies (Møller et al., 2008).

Two other items were used for a global assessment of subjective wellbeing: one inquired about the past 30 days (which was the period after the term exams). This item reads “*If you were to express how you felt during the past 30 days on a scale ranging from 0 to 10 (where 0 stands for “I am very dissatisfied with my life,” and 10 is “I am very satisfied with my life”), what number would you pick?*” A similar second item inquired about satisfaction with life during COVID-19.

As one goal of this study is to assess the effects of COVID-19 on subjective wellbeing, we need pre-COVID-19 measures of subjective wellbeing to compare with. We could obtain some figures that we will use for this sake. Most recent are valid ACSA scores obtained from 1,078 students in Economics (response rate 17.6%; of 6,139 students, of whom 52.1% female; 36.2% under 21 years old; 59.3% 21–25 years; and 4.4% >26 years) at the Ghent University in early 2020, before Belgium imposed a first lockdown to deal with the COVID-19 pandemic (Mean = 1.50; SD = 2.49; Verlet, personal communication, 2021; Verhaeghe et al., 2020). In 2014 and 2015, respectively, 80 and 118 Belgian students reported ACSA scores with means 1.77 and 1.87 and SDs 2.18 and 1.78 (Verhofstadt, personal communication, 2021), where the figures from 2014 were extracted from the data reported in Verhofstadt et al. (2019). For a single item assessment of subjective wellbeing on a 0-to-10 scale, we obtained some figures from “World Data Base of Happiness” of Veenhoven (2020). Here we found that on a 0–10 scale, wellbeing measures can be expected roughly around 6.65–7.7 with SDs between 1.4 and 2.0 (Michalos, 1991; Van Ee and Van Dijk, 2005; Van Dongen and Van Der Graaf, 2012; OECD, 2017; Veenhoven et al., 2021). More recent SWB sores obtained from Belgian students were reported by De Coninck et al. (2019). Interestingly, in this latter study, it was found that SWB in students had dropped from mean = 7.1 (SD = 1.60) at the start of the academic year to mean = 6.9 (SD = 1.63) at the end of the first semester. This latter figure was found at the same period of the year as the period where our data have been collected. Therefore, we will use this average for comparison with our data collected during COVID-19. Note that in the latter study, between summer 2020 and spring 2021, in the general Dutch population a drop in average SWB, from 6.89 to 6.74 was observed (Veenhoven et al., 2021).

#### Psychological distress

The six-item Kessler Screening Scale for Psychological Distress (K6; Kessler et al., 2002) is a dimensional measure of non-specific psychological distress. The questionnaire has been used extensively in community epidemiological surveys and was found a valid measure with excellent internal consistency and reliability (Cronbach’s alpha = 0.89; Kessler et al., 2002) to assess current emotional distress and to screen for the presence of non-specific mental disorders (Kessler et al., 2003, 2010). In the current sample, the K6-scale has demonstrated a good Cronbach’s alpha (α = 0.81). Using a 30-day reference period, respondents are required to rate (on a five-point rating scale that ranged from 0 = none of the time to 4 = all the time) how often they felt nervous, hopeless, restless, or fidgety, so sad that nothing could cheer them up, that everything was an effort and worthless. Higher (summated) scores indicate more psychological distress (range 0–24).

Generally, K6-scores show a right skew (Tomitaka et al., 2019) across different age groups; both in adolescent (Green et al., 2010; Peiper et al., 2014) and adult samples (Kang et al., 2015; Tomitaka and Furukawa, 2021). The majority of respondents report minimal or minor psychological distress (K6 score up to 4).

Approximately, 50% of adolescents get K6-scores between 0 and 3 (Green et al., 2010; Peiper et al., 2014). More specifically, Green et al. (2010) report the following distribution: 0 (24.1%), 1 (10.3%), 2 (8.6%), and 3 (7.1%). Mean K6 scores seem slightly higher in transitioning youth samples (including college students); about half (53.4%) of the undergraduates in a Chinese study scored 4 or lower. Proportions of those who scored “0,” “1,” “2,” “3,” and “4” were 5.6, 8.5, 13.6, 13.8, and 11.8%, respectively (Kang et al., 2015). A similar proportion of 18–25-year-old reported minor psychological distress (scores ≤4) in two American studies—respectively 50.5% (Prochaska et al., 2012) and 55% (Shafer et al., 2017). In a comparable sample of Dutch university freshmen students, Dopmeijer et al. (in prep) found average of 11.4 (SD = 4.4; 2014 cohort) and 11.5 (SD = 4.6; 2015 cohort).

The standard cutoff score of 13 or higher on the K6 is applied to identify persons with (non-specific) serious mental distress (referring to people with a high likelihood of having a diagnosable mental illness severe enough to cause functional limitations and to require treatment; Kessler et al., 2003). Scores between 5 and 12 are referred to as “moderate mental distress,” while scores of 13 and higher are referred to as “serious mental distress.” Based on a large-scale study among the general population in California, Prochaska et al. (2012) found 13.6% of the young people between 18 and 25 met the criteria for serious mental distress (K6 ≥ 13) and another 35.9% met the criteria for moderate mental distress (5 ≤ K6 ≤ 12). Shafer et al. (2017) found the group of students likely to have serious mental distress somewhat smaller (9% with K6-scores ≥13), and 36% with moderate mental distress. Conversely, the mean K6-scores among Canadian student-athletes were 8.2; 19.8% of that sample met the criteria for serious mental distress (K6 ≥ 13; Sullivan et al., 2019).

### Procedure

As indicated above, this study is part of an initiative from the university to give a warm support to all students with a personal phone call during the COVID-19 pandemic. As it is uncommon to contact students on their private phone, the procedure included several possibilities for students not to take part (see Figure 1). Note that non-bachelor or non-master students (e.g., PhD students and post graduates) were excluded from this study as those typically are in a quite different social situation, often with a family of their own etc.

First, all 15,475 bachelors and master students were informed about the project *via* the VUB newsletter & social media. Those who did not opt out at that time were sent an email to announce they would be called soon, unless they replied not wanting to participate. Next, the remaining students were called.

At the start of the phone interview, the called students’ active informed consent was additionally obtained. Upon verbal consent (*n* = 7,154), students were asked to provide their age, gender, and year of study, before completing the interview. The interviews were administered following an interview guide, with a major focus on providing students a compassionate response to the subjective COVID-19-related malaise and suffering. The average duration of the interview was 15 min.

The interviews were performed by 134 first and second year master students in clinical psychology who followed a 2-h online training, specifically designed for the present interview, supplemented by weekly supervisions. The training comprised how to make a warm and caring connection, how to administer the brief structured measures (as mentioned above) and adequately respond to persons in distress or when confronted with a risk/acute situation. A team of experienced clinical psychologists provided in a permanence system, ensuring a continuous backup for the master students. Interventions as follow-up after the phone call were based on the responses on the K6 questionnaire. Four levels of psychological distress and associated interventions were differentiated. First, students with no or very little psychological distress (K6 lower than 5) were referred to a general information website of the university to keep up to date with regard to financial, social, and mental support. Second, students with mild psychological distress (K6 between 5 and 12 points) where referred to the student psychologist services on campus, online peer-support groups, or national hotlines. Third, those who scored above the K6 cutoff (K6 ≥ 13) were referred to external psychologists and mental health services. Fourth, those who answered high on the K6 item4 “worthless; hopeless and feelings of depression” and/or showed signs of an acutely disturbed sense of reality/aggression/suicidality or overwhelming emotionality were handled as potential acute risk and advised to contact crisis services (e.g., emergency department) or their GP, and were contacted by our team of licensed clinical psychologists within 7 days for follow-up, after the students’ explicit consent.

The study was approved by the Medical Ethics Committee of UZ Brussels (B.U.N. 1432021000383).

### Data analysis

Statistical analyses were performed in R version 4.1.2 (R Core Team, 2021), using the ggstatsplot package (Patil, 2021). To test differences between groups, we used Welch’s *t*-test, Welch’s ANOVA, and Pearson’s Chi^2^, with Hedges’ *g*, omega^2^, and Cramer’s V effect sizes, respectively. For each effect size, 95% CIs are reported. Next to these inferential statistics, we also report their Bayesian counterparts. The Bayes Factor can be used to assess the evidence in favor of the null hypothesis as opposed to the alternative hypothesis (BF_01_ > 4.61 = decisive evidence for H_0_, 3.40 < BF_01_ < 4.61 = Very strong evidence for H_0_, 2.30 < BF_01_ < 3.40 = Strong evidence for H_0_, 1.10 < BF_01_ < 2.30 = Substantial evidence for H_0_, −1.10 < BF01 < 1.10 = Not worth more than a bare mention, −2.30 < BF_01_ < −1.10 = Substantial evidence for H_1_, −3.40 < BF_01_ < −2.30 = Strong evidence for H_1_, −4.61 < BF_01_ < −3.40 = Very strong evidence for H_1_, and BF_01_ < −4.61 = Decisive evidence for H_1_).

## Results

### Subjective wellbeing

**Figure 2:**
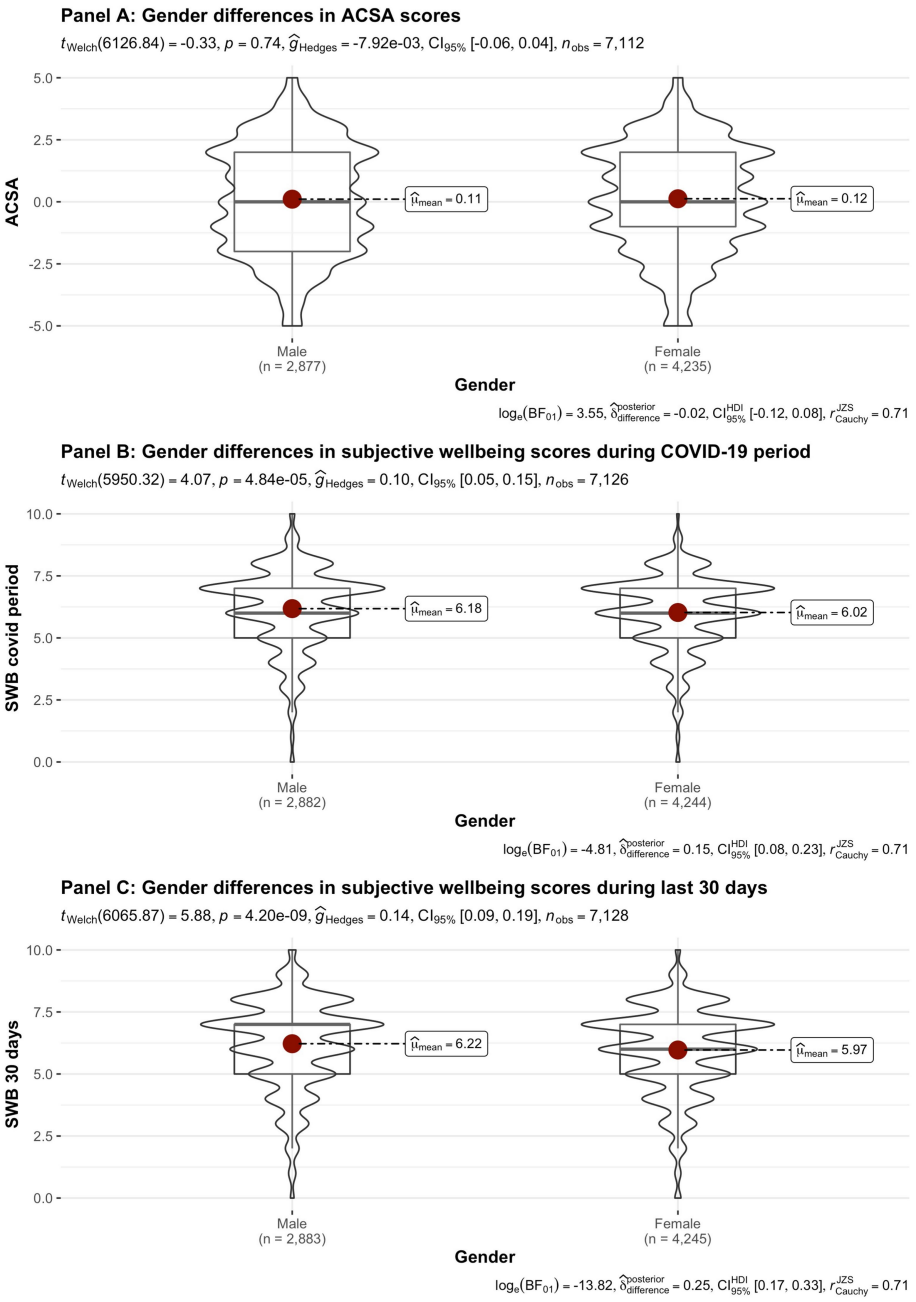
Mean Anamnestic Comparative Self-Assessment (ACSA; during the last 2 weeks, on a −5 to +5 scale) and subjective wellbeing (SWB)-scores (during the COVID-19 period, and during the past 30 days, both on a 0–10 scale) comparing student groups during COVID-19 in terms of gender.

**Figure 3:**
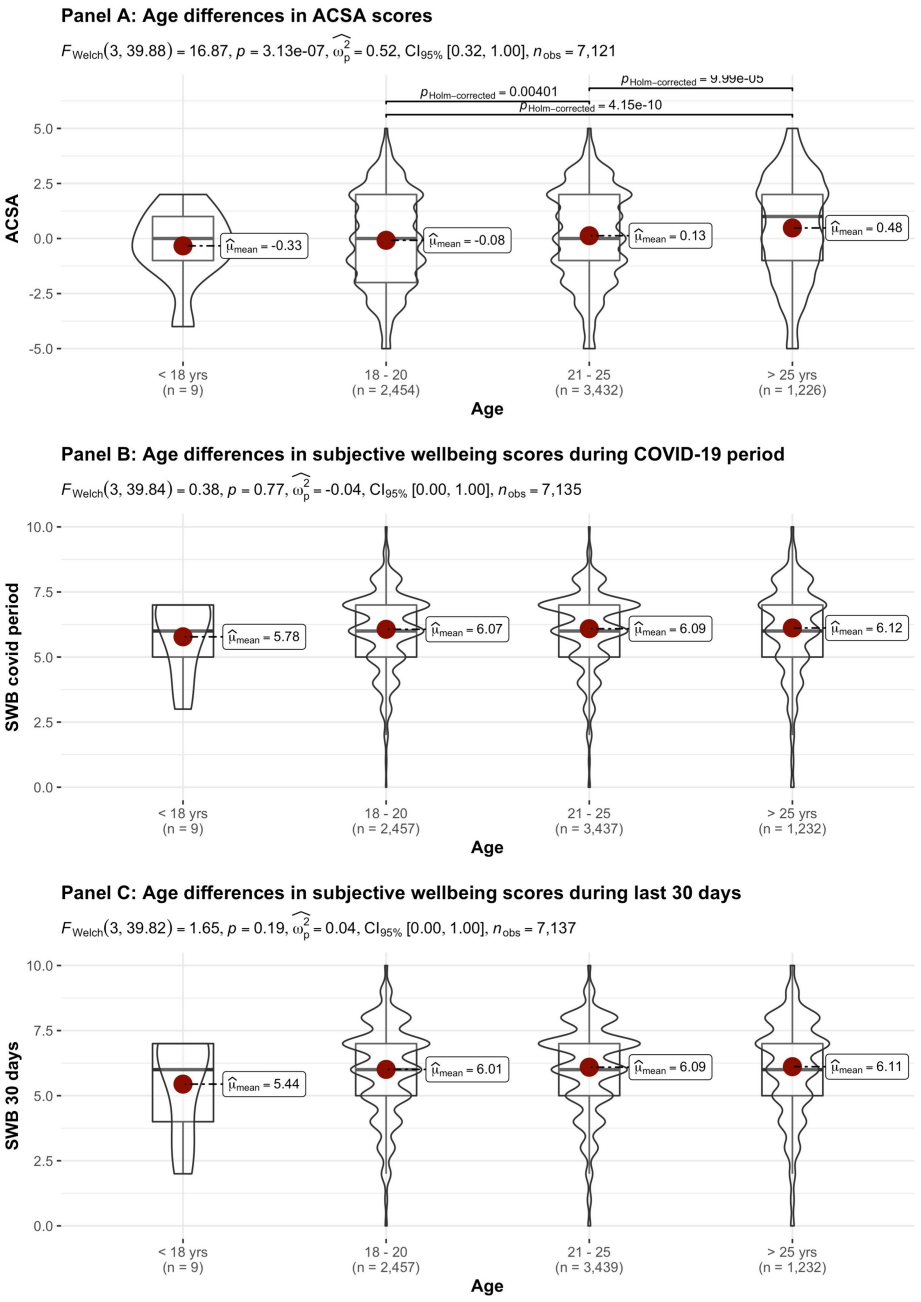
Mean ACSA (during the last 2  weeks, on a −5 to +5 scale) and SWB-scores (during the COVID-19 period, and during the past 30 days, both on a 0–10 scale) comparing student groups during COVID-19 in terms of age.

**Figure 4:**
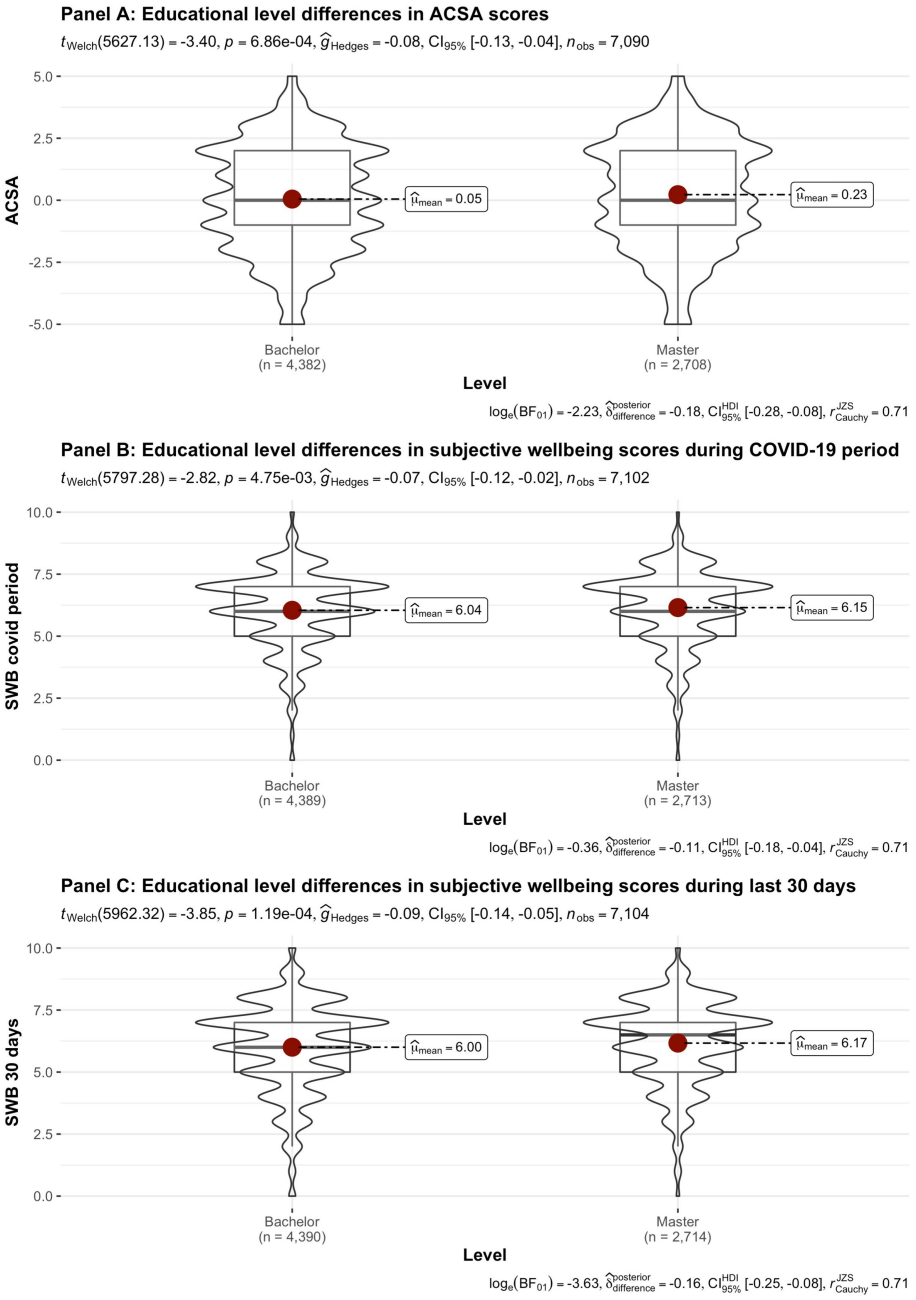
Mean ACSA (during the last 2 weeks, on a −5 to +5 scale) and SWB-scores (during the COVID-19 period, and during the past 30 days, both on a 0–10 scale) comparing student groups during COVID-19 in terms of educational level.

**Figure 5:**
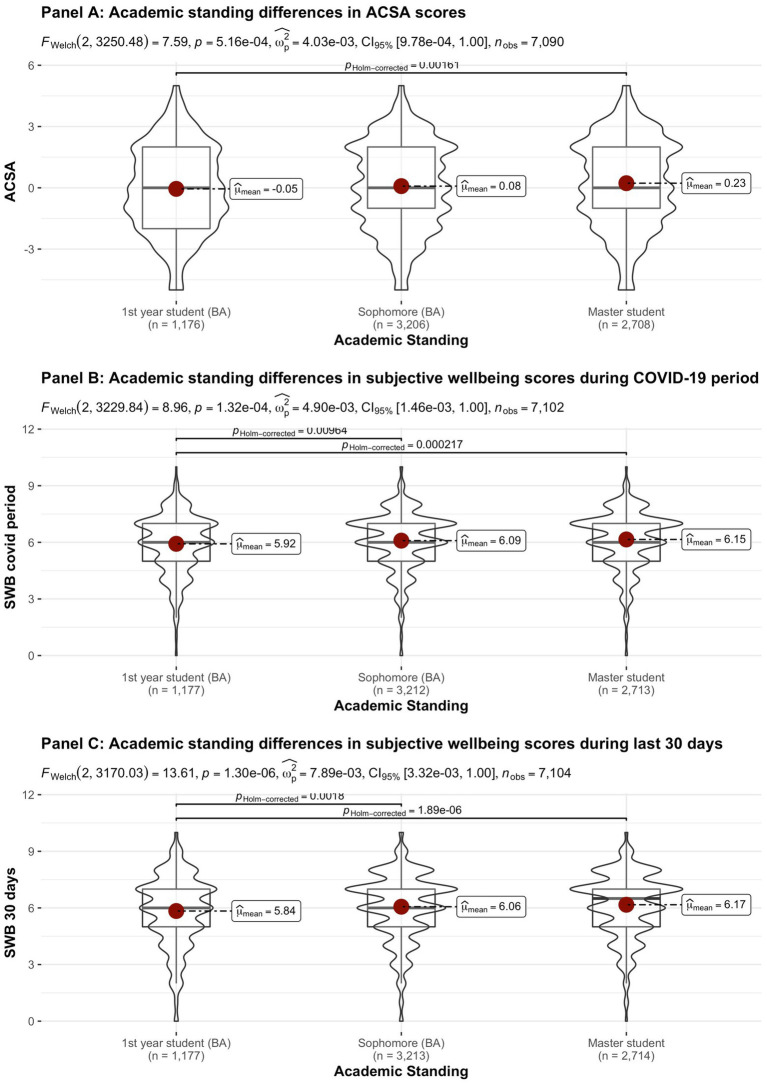
Mean ACSA (during the last 2 weeks, on a −5 to +5 scale) and SWB-scores (during the COVID-19 period, and during the past 30 days, both on a 0–10 scale) comparing student groups during COVID-19 in terms of academic standing.

**Figure 6:**
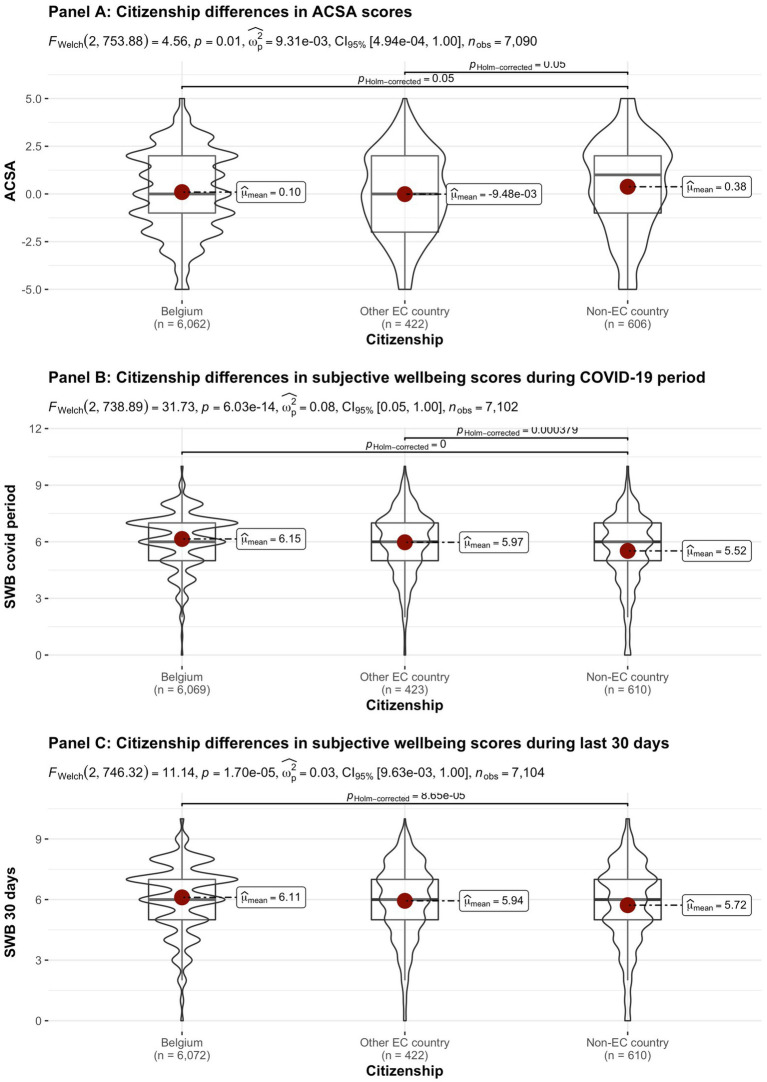
Mean ACSA (during the last 2 weeks, on a −5 to +5 scale) and SWB-scores (during the COVID-19 period, and during the past 30 days, both on a 0–10 scale) comparing student groups during COVID-19 in terms of citizenship.

**Figure 7:**
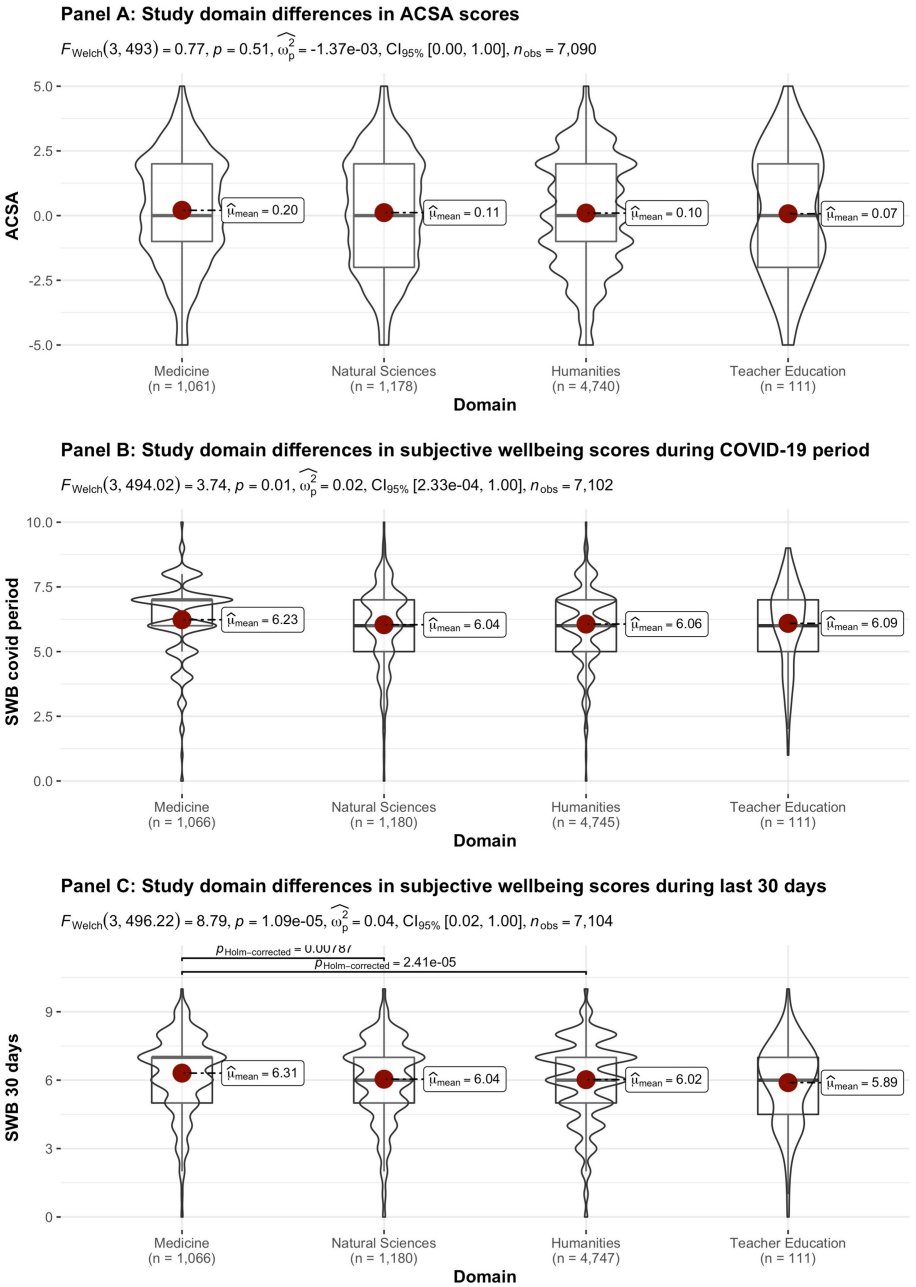
Mean ACSA (during the last 2 weeks, on a −5 to +5 scale) and SWB-scores (during the COVID-19 period, and during the past 30 days, both on a 0–10 scale) comparing student groups during COVID-19 in terms of study domain.

**Figure 8:**
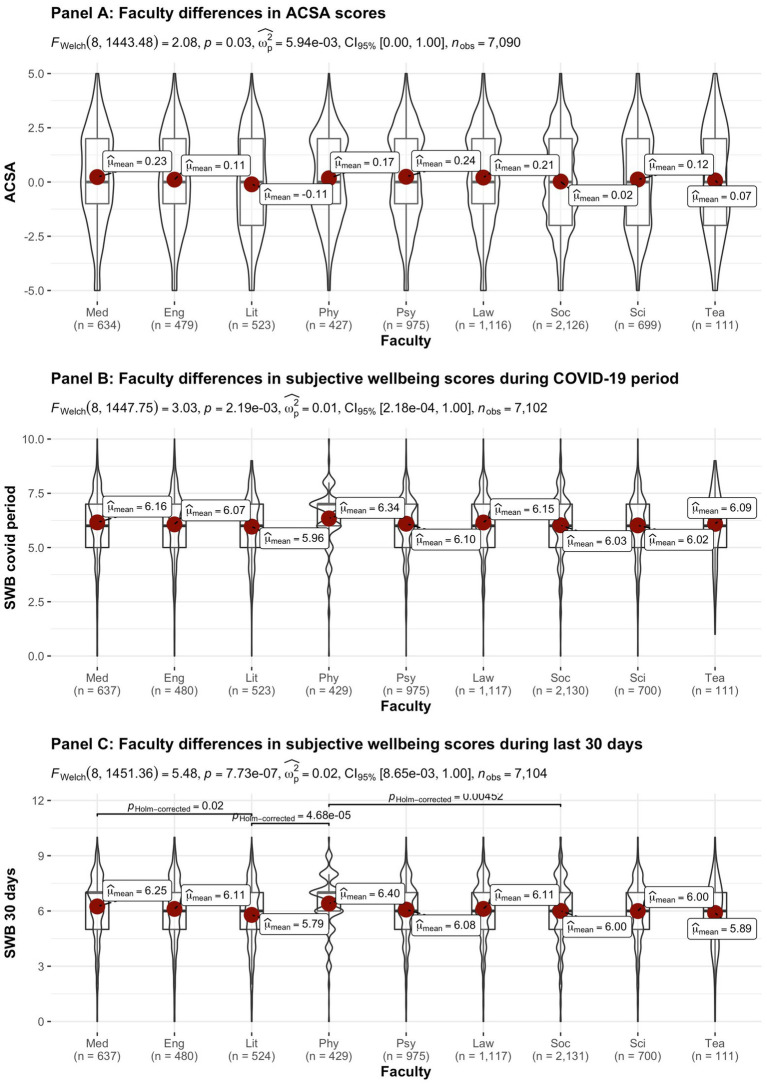
Mean ACSA (during the last 2 weeks, on a −5 to +5 scale) and SWB-scores (during the COVID-19 period, and during the past 30 days, both on a 0–10 scale) comparing student groups during COVID-19 in terms of faculty. Med, Medicine and Pharmacy; Eng, Engineering Sciences; Lit, Literature and Philosophy; Phy, Physical Education & Physiotherapy; Psy, Psychology and Educational Sciences; Law, Law and Criminology; Soc, Social Sciences and Business School; Sci, Sciences and Bio-engineering; and Tea, Teacher Education.

Figures 2–8 provide an overview of the results regarding SWB. Although in some cases significant differences in SWB were observed between subsamples [e.g., gender (men reported higher SWB), academic standing (Bachelor students reported lower SWB compared to Master students; freshmen had the lowest scores, Master students the highest), citizenship (non-EC students had higher scores), and faculties and domains of science (students from the Literature and Philosophy faculty reported the lowest scores vs. students from the Medicine and Pharmacy faculty reported the highest scores)], these differences are all quite small (smaller than 1 scale point) and have small effect sizes, suggesting that differences between subsamples are relatively minor and students overall suffered from COVID-19.

On average, the current sample of students reports significantly lower levels of subjective wellbeing (average ACSA = 0.12, *SD* = 2.15, *N* = 7,121) than a comparable group of students at another Flemish university that was inquired at a similar time of the year exactly 1 year earlier (average ACSA = 1.50, *SD* = 2.49, *N* = 1,078, of whom 52.1% female; Verlet, personal communication 2021; Verhaeghe et al., 2020), which is supported by an independent samples *t*-test [*t* (7,120) = −54.44, *p* < 0.001, Hedges’ *g* = −0.65, CI_95%_ = [−0.69, −0.60]]. Similarly, mean SWB scores in our sample, both during COVID-19 (*mean* = 6.09, *SD* = 1.57) and during the past 30 days (*mean* = 6.07, *SD* = 1.78) were significantly lower [independent samples *t*-tests with *t* (7,134) = −43.76, *p* < 0.001, Hedges’ *g* = 2.92, CI_95%_ = [2.85, 2.98] and *t* (7,136) = 6.-39.61, *p* < 0.001, Hedges’ *g* = 2.57, CI_95%_ = [2.51, 2.63], respectively] than SWB ratings obtained in a Belgian sample of university students in 2019, right after the first semester of the academic year (*mean SWB* = 6.90, *SD* = 1.63, *n* = 194; De Coninck et al., 2019).

Noteworthy, small differences in the three measures of subjective wellbeing were found between the different faculties, which were however statistically significant (see Figure 8C). While differences between faculties were statistically significant for SWB during the COVID-19 pandemic, the Bayes factor gave decisive evidence for the null hypothesis that there are no differences between faculties. In contrast, the Bayes factor indicated substantial evidence for differences between faculties for SWB during the past 30 days. However, the differences in SWB during the past 30 days observed across faculties are relatively small when compared to the significant drop in all SWB measures during COVID-19, as inferred from the comparisons of our data to pre-COVID-19 data collected in similar samples at similar periods during the academic year (end first semester; De Coninck et al., 2019; Verhaeghe et al., 2020). It is remarked that the “drop” in ACSA-ratings is clearly greater than in the other SWB measures, which corresponds to the expectancy that ACSA can overcome a response shift.

### Psychological symptoms

**Figure 9:**
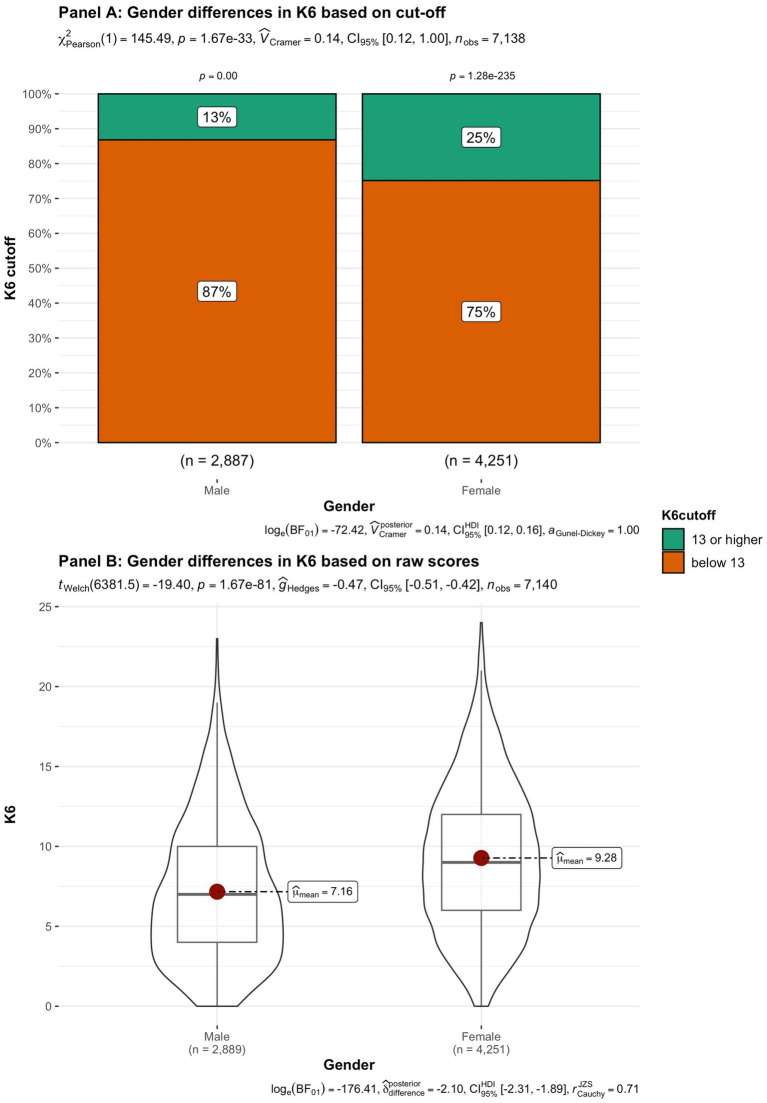
Summated scores on the K6 (indicator of mental illness and considered as a cutoff variable) across student subgroups in terms of gender.

**Figure 10:**
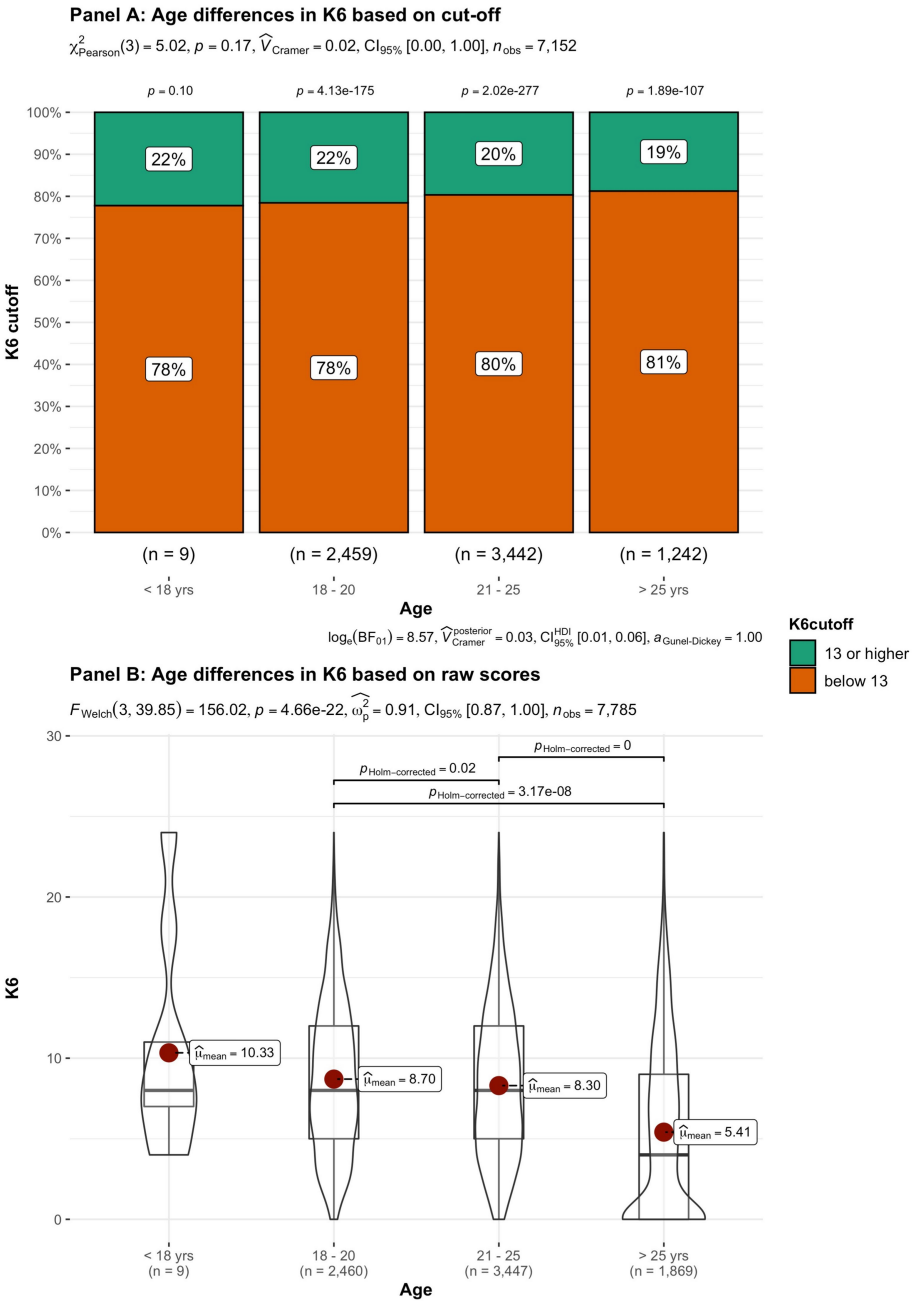
Summated scores on the K6 (indicator of mental illness and considered as a cutoff variable) across student subgroups in terms of age.

**Figure 11:**
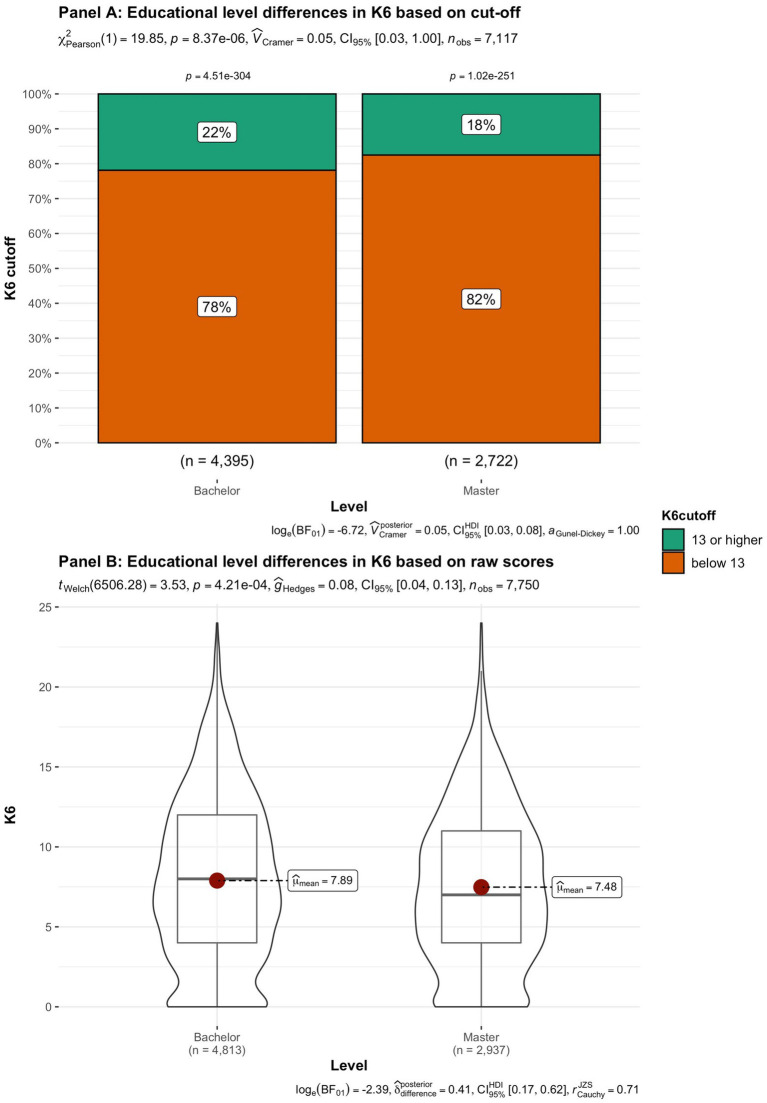
Summated scores on the K6 (indicator of mental illness and considered as a cutoff variable) across student subgroups in terms of educational level.

**Figure 12:**
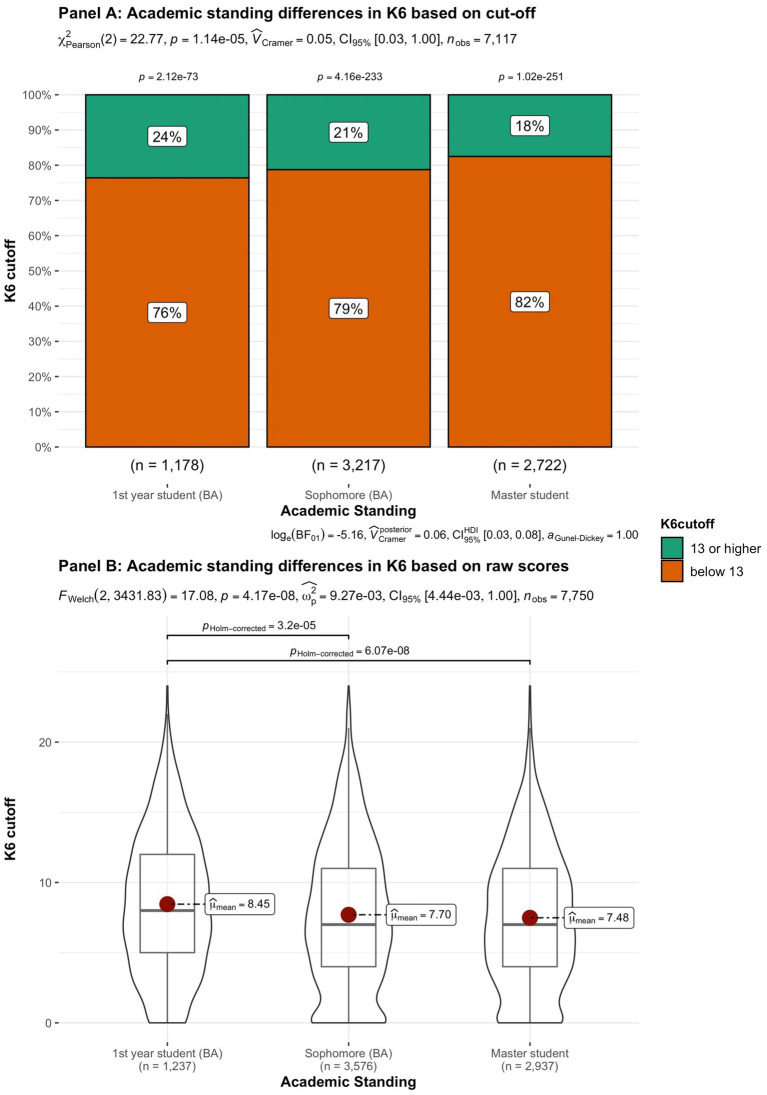
Summated scores on the K6 (indicator of mental illness and considered as a cutoff variable) across student subgroups in terms of academic standing.

**Figure 13:**
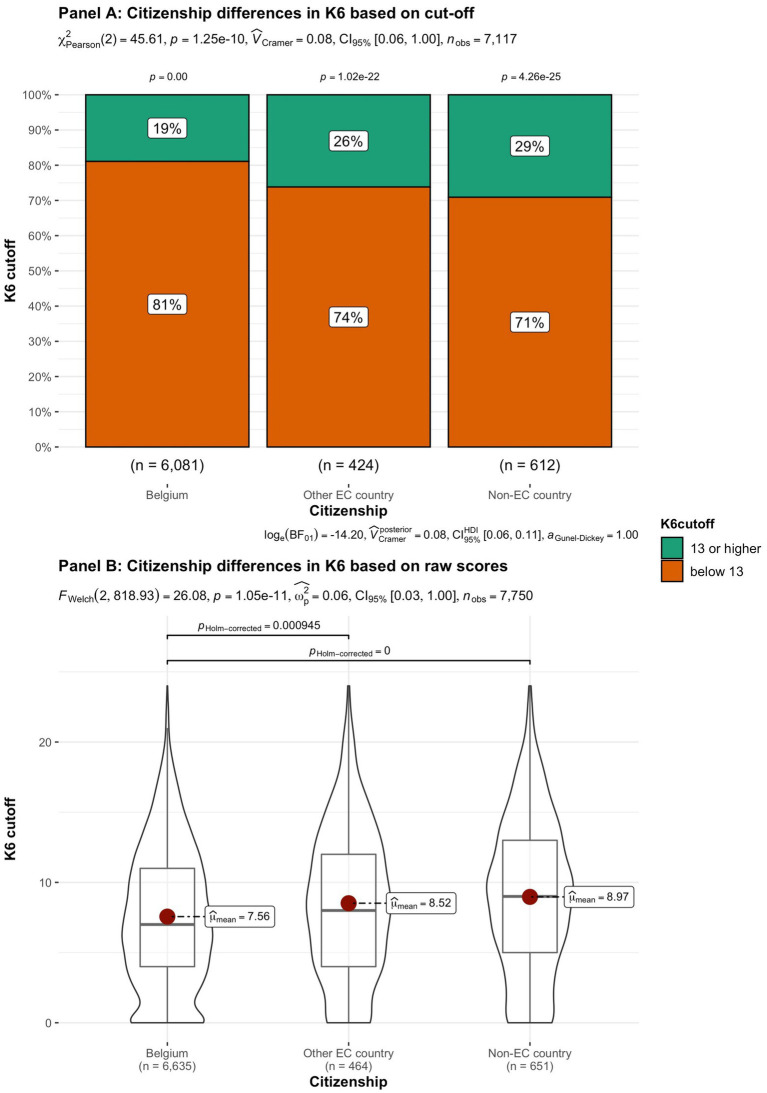
Summated scores on the K6 (indicator of mental illness and considered as a cutoff variable) across student subgroups in terms of citizenship.

**Figure 14:**
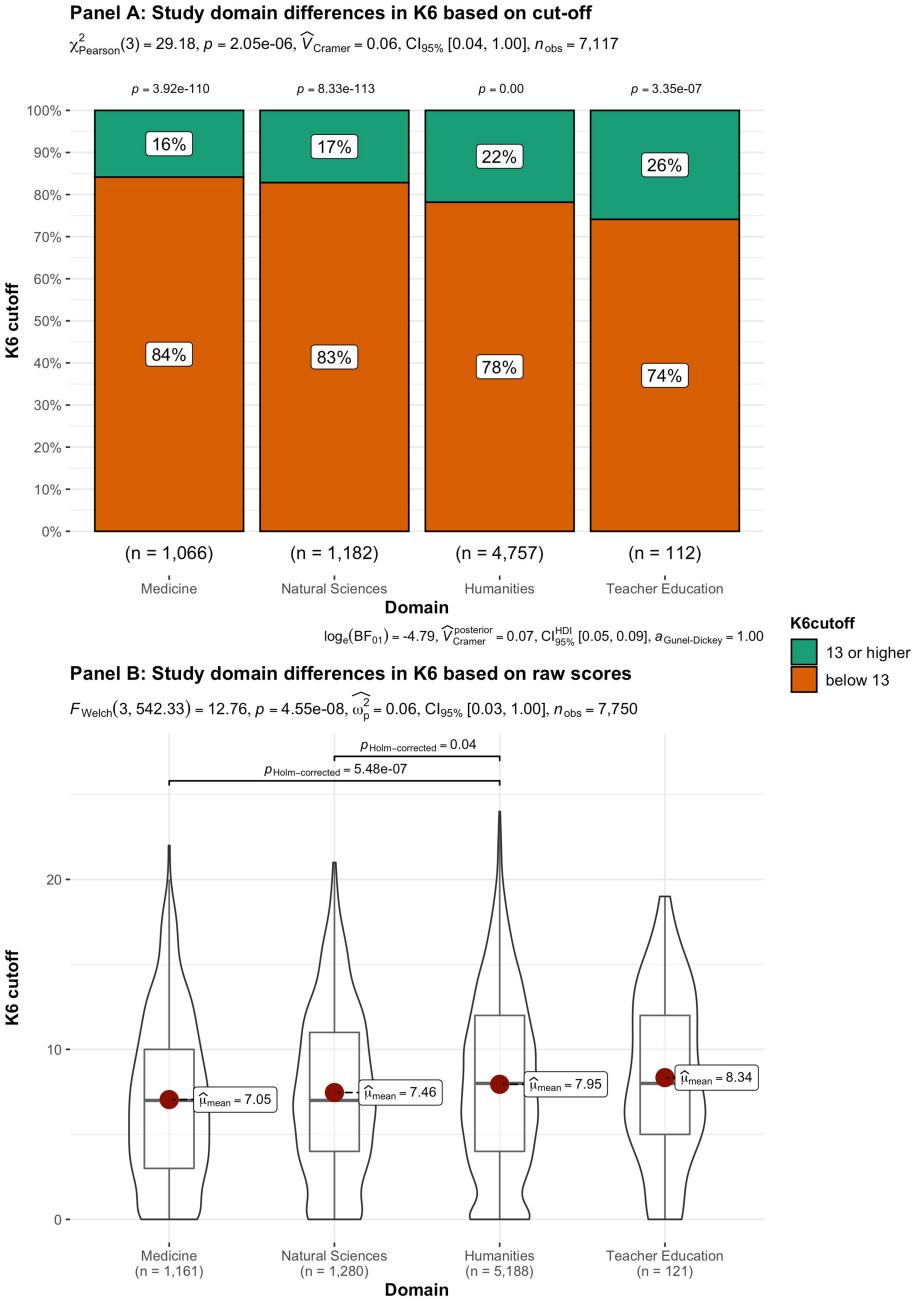
Summated scores on the K6 (indicator of mental illness and considered as a cutoff variable) across student subgroups in terms of study domain.

**Figure 15:**
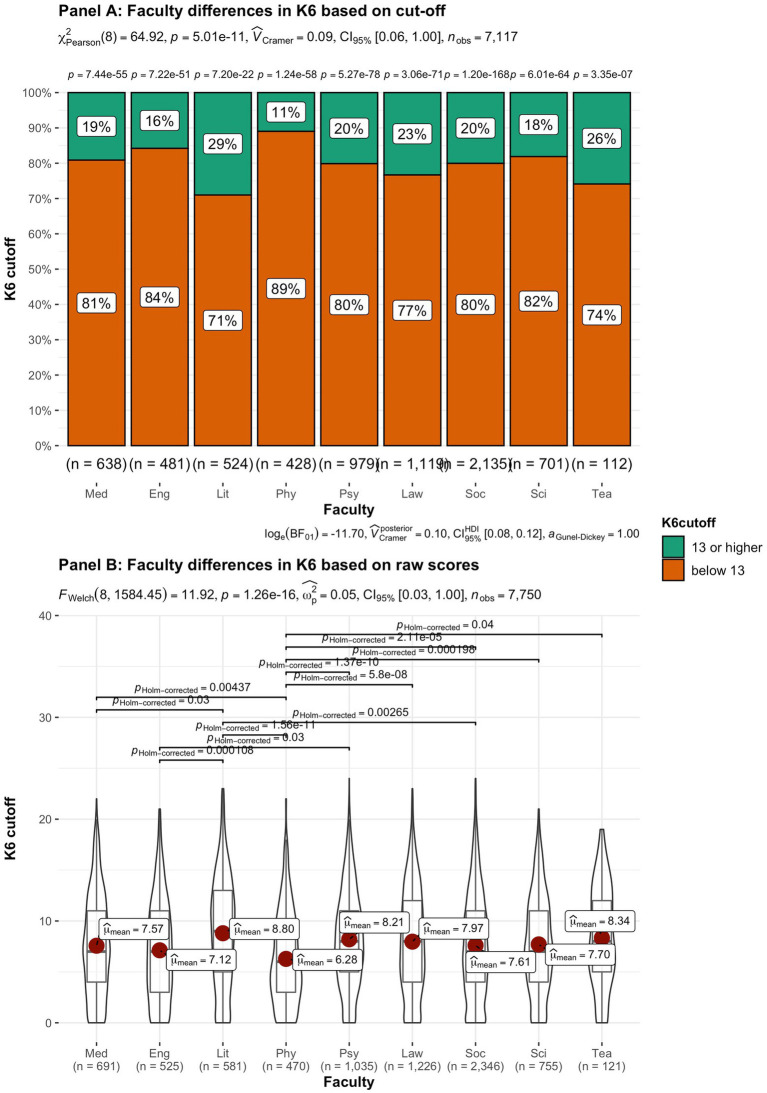
Summated scores on the K6 (indicator of mental illness and considered as a cutoff variable) across student subgroups in terms of faculty. Med, Medicine and Pharmacy; Eng, Engineering Sciences; Lit, Literature and Philosophy; Phy, Physical Education and Physiotherapy; Psy, Psychology and Educational Sciences; Law, Law and Criminology; Soc, Social Sciences and Business School; Sci, Sciences and Bio-engineering; and Tea, Teacher Education.

In addition to subjective wellbeing, psychological distress was compared in the student groups. In Figures 9–15 the occurrence of signals of mental illness (K6 total score ≥ 13) versus normal (K6 below 13) is presented for the same student groups as in the comparison for subjective wellbeing in Figures 2–8. Overall, 20.16% of all students displayed signals of mental illness (K6 total score ≥ 13), while the remaining 79.84% reported normal scores. Compared to a Dutch sample of fulltime freshman students surveyed in 2014 and 2015 (Dopmeijer et al., in prep), our sample scored significantly lower on the K6 scale [compared to 2014: *t* (7,784) = −64.19, *p* < 0.001, Hedges’ *g* = −0.73; compared to 2015: *t* (7,784) = −65.95, *p* < 0.001, Hedges’ *g* = −0.75]. Freshmen, bachelor students (especially freshmen), female students, younger students, non-Belgian students, and students studying human sciences were more at risk for increased levels of psychological symptoms.

## Discussion

In the current study, the prevalence and severity of psychological symptoms and subjective wellbeing of Belgian university students was examined during the COVID-19 pandemic. In addition, the association with factors such as gender, academic standing, and age was examined.

In line with earlier research (e.g., Savage et al., 2020) significantly lower levels of subjective wellbeing, compared to norms from comparable student population’s pre-COVID, were found for all three measures of subjective wellbeing. Generally speaking, students experienced their life during the pandemic as less satisfying compared to their life before COVID-19.

More specifically, differences in wellbeing across subgroups of students were small, meaning that overall students have suffered from COVID-19, and that all subgroups of students were affected. When comparing subjective wellbeing during the pandemic to one’s own life with the ACSA, by taking the best and the worst periods in one’s own life to substantiate the meaning of the scale ends, the decrease in subjective wellbeing is more pronounced (i.e., larger effect size) than with both other global assessments of subjective wellbeing. This suggests that when students compare their current subjective wellbeing with other periods in their life, the impact of the pandemic and the measures to contain becomes most conspicuous.

However, when asked to rate their subjective wellbeing without reference to their own life, the effect of the pandemic and the measures is somewhat smaller. When assessing one’s wellbeing, peer relativity (the observation that one is not worse off than their peers) may partly dampen the expression of the experienced drop in personal wellbeing, and so result in less contrasting outcome measures.

About 20% (*n* = 1,535) of students scored 13 or higher on the K6. This score suggests that one in five students are likely to experience significant levels of psychological distress at the time of the interview, requiring assessment or treatment. Although the K6 measure-like some other screening instruments-is not diagnostic, it is generally considered to be able to discriminate psychiatric cases from non-cases (Furukawa et al., 2003; Kessler et al., 2003; Green et al., 2010). Consequently, the high number of students with a score above the cutoff attracts our attention. Comparing the results of our study with the percentages found in earlier studies [13.6% (Prochaska et al., 2012), 9% (Shafer et al., 2017), and 19.8% (Sullivan et al., 2019)] with this instrument, this number is rather elevated, and in line with the findings of Essau and de la Torre-Luque (2021) during the COVID pandemic. However, this number is low compared with the numbers (65.49%, Rens et al., 2021) found in a Belgian sample of 16–24-year-old, or in a United Kingdom-study investigating the mental health status of university students at an early stage of the pandemic (53.4% above the clinical cutoff for depression and 51.5% above the anxiety cut off; Chen and Lucock, 2022). The timing of the survey (March 2021 vs. April 2020) and the use of different instruments and cutoff points may explain these disparities in prevalence of psychopathology. In line with Patel et al. (2022), based on a meta-analysis of 11 longitudinal studies, females are particularly at risk for heightened psychological distress during the COVID-19 pandemic. Also in line with previous studies (e.g., Chen et al., 2020; Kecojevic et al., 2020; Rens et al., 2021), freshmen, non-Belgian and students studying human sciences were found to be more at risk for increased levels of psychological symptoms. Consequently, our findings suggest that the profile of these students, who are more at risk for serious mental distress, is characterized by a lack of experience, uncertainty, and immature social networks. These students seem more vulnerable to the threats of COVID-19 and for measures such as the suspension of classes, closure of the campus, the shift from in-person to online teaching, and disrupted academic prospects, while travel restrictions and social and physical distancing measures affected the interactions and social networks and life of these students. However, as we do not have any information about psychological distress or pre-existing mental health difficulties before the COVID-19 pandemic, these possible explanations remain speculative. Conversely, this profile may explain the relatively limited rise in psychological distress at the group level. Indeed, more than 85% of our sample were Belgian students with the ability to return home, which may have mitigated the negative effects of the confinement (Cao et al., 2020).

Still, differences between subgroups remain rather limited. No matter what subgroup a student belongs to, (group) levels of mental distress remain high. Although we can consider these increased numbers as a normal and understandable reaction to an adverse situation, they still are striking. It might be useful to take the fluctuation of symptoms over time into consideration. For example, Saunders et al. (2021) identified four different trajectories of depression and anxiety symptoms over time during the pandemic (before, during, and after lockdown) in the general population. Nearly 30% of participants experienced trajectories with symptoms in the clinical range during lockdown, and more importantly, did not follow the average curve (likely symptom trajectory) or majority group. This kind of process information is highly informative for providing tailor-made support to reduce the likelihood of longer-term problems in university students.

Mental health of higher education students has already been a concern in the years before the COVID-19 pandemic; it affects student engagement and dropout. Data from continuous surveys and assessments of all students are required to help universities address this issue. These data will not only be helpful in identifying the needs of students but will also support the fostering of a campus culture that prioritizes wellbeing and mental health as a value.

## Limitations

Notwithstanding the strengths of this project (a naturalistic design, caring approach), large N, and robust findings in line with previous studies, there are several notable limitations as well. First, the response rate of 50% was rather low, which is often the case in large-scale college student surveys (e.g., Karyotaki et al., 2020). Although, we are not aware of a systematic bias, low response rates can threaten generalizability of the results to all students. Although, we seem to have targeted a representative sample of VUB bachelor and master students in terms of gender, age, nationality, and faculties, we must be careful in interpretation of results, due to potential self-selection biases. It might be that those students who already experienced low academic motivation, or detachment from the university, or severely depressed were less likely to pick up their phones. Thirdly, because this study was done during the pandemic, no “baseline” could be established for the student population of our university, and we had to rely on reference data from other young adults and universities. Finally, a screening is not equal to a diagnosis, and elevated levels of psychological symptoms can describe a wide range of conditions not necessarily equaling a mental disorder.

## Implications

Campion et al. (2020) state a triple global public mental health challenge of the COVID-19 pandemic: (1) to prevent an associated increase in mental disorders and a reduction in mental wellbeing across populations; (2) to protect people with a mental disorder from COVID-19, and the associated consequences, given their increased vulnerability; and (3) to provide appropriate public mental health interventions to health professionals. The first two challenges are transferrable to the university student population and refer to preventive and interventional measures during the pandemic. The aims of our project “The university cares for you,” during which the data of the current study were collected, addresses these challenges by: (1) making a compassionate, caring telephonic contact with each VUB student in order to give them courage, recognition, and hope, (2) examining the current wellbeing of our VUB students, (3) estimating the number of students with poor subjective wellbeing, and (4) detecting students at risk for or with mental health problems and refer them to appropriate mental health services.

Universities and colleges should invest in both a mental health promoting environment (Reis et al., 2018) as well as in increasing mental health literacy and may, e.g., provide low-threshold online interventions promoting resilience, needs crafting, help-seeking (e.g., www.moodspace.be) for all students, and targeted evidence-based group trainings for at risk students (Kohls et al., 2021). Group trainings targeting transdiagnostic mechanisms, such as mindfulness (Dawson et al., 2020), self-compassion training (Bluth and Neff, 2018), or emotion regulation skills training (Southward et al., 2021), can help mitigate the rise of psychological symptoms, and increase the subjective wellbeing and resilience of university students.

Also, previous studies (e.g., Kleiman et al., 2020; Hamza et al., 2021) have shown the importance of low social support, lack of indirect social contact with peers, and feelings of loneliness as important mediators in heightened risk for psychological symptoms and decrease in subjective wellbeing during the COVID-19 pandemic. Therefore, universities should invest in activities nurturing direct social interactions between students. Next to the need for relatedness, Vansteenkiste and Ryan (2013) underscore the basic psychological needs for autonomy and competence as essential ingredients for a positive subjective wellbeing and a healthy psychological development. Higher education can actively promote improving need satisfaction, with on campus/online programs for needs crafting (Laporte et al., 2021) with for example LifeCraft, a seven-session (online) program that promotes individuals’ proactive attempts to uplift their need-based experiences (i.e., need crafting).

## Conclusion

Although COVID-19 does have a detrimental impact on the subjective wellbeing in our student population, the majority of university students seem to be coping adequately in these adverse times. Moreover, although levels of mild psychological symptoms have increased, our study does not reveal an significant increase in severe psychological distress. Our results underscore the necessity for universal mental health prevention and need for psychological support for students. Furthermore, female students, freshmen and international students are at heightened risk for severe psychological distress and can be at-risk group for targeted interventions.

## Data availability statement

The raw data supporting the conclusions of this article will be made available by the authors, without undue reservation.

## Ethics statement

The studies involving human participants were reviewed and approved by Medisch Ethische Commissie—VUB. Written informed consent for participation was not required for this study in accordance with the national legislation and the institutional requirements.

## Author contributions

IB coordinated the project and wrote the intro and discussion section. JM followed up the daily coordination. JV wrote the discussion and followed up the process of the project. TV and PT were responsible for the analyses. VS and CS co-wrote the introduction together with IB and assisted in the setup of the research protocol. JV assisted IB in the discussion part and follow-up. All authors contributed to the article and approved the submitted version.

## Funding

This work was funded by Vrije Universiteit Brussel, Department of student affairs (project: VUB Geeft om Jou).

## Conflict of interest

The authors declare that the research was conducted in the absence of any commercial or financial relationships that could be construed as a potential conflict of interest.

## Publisher’s note

All claims expressed in this article are solely those of the authors and do not necessarily represent those of their affiliated organizations, or those of the publisher, the editors and the reviewers. Any product that may be evaluated in this article, or claim that may be made by its manufacturer, is not guaranteed or endorsed by the publisher.
